# Contrasting the population genetic structure of a specialist (*Hexaglandula corynosoma*: Acanthocephala: Polymorphidae) and a generalist parasite (*Southwellina hispida*) distributed sympatrically in Mexico

**DOI:** 10.1017/S0031182023000033

**Published:** 2023-04

**Authors:** Martín García-Varela, Alejandra López-Jiménez, Marcelo Tonatiuh González-García, Ana Lucia Sereno-Uribe, Leopoldo Andrade-Gómez

**Affiliations:** 1 Departamento de Zoología, Instituto de Biología, Universidad Nacional Autónoma de México (UNAM), Avenida Universidad 3000, Ciudad Universitaria, CP 04510 México City, Mexico; 2 Posgrado en Ciencias Biológicas, Universidad Nacional Autónoma de México, Avenida Universidad 3000, Ciudad Universitaria, CP 04510 México City, Mexico; 3 Escuela Nacional de Estudios Superiores Unidad Mérida, Km 4.5 Carretera Mérida-Tetiz, Ucú, Yucatán CP 97357, Mexico

**Keywords:** Acanthocephalans, generalist, Mexico, Polymorphidae, phylogeography, specialist

## Abstract

Polymorphidae is a monophyletic group of acanthocephalans distributed worldwide. Within this family, *Hexaglandula corynosoma* is a specialist species that uses a single bird species as a definitive host. *Southwellina hispida* is a generalist species that uses a broad spectrum of definitive hosts to complete its life cycle. In the current research, sequences of cytochrome c oxidase subunit 1 (*cox1*) from mitochondrial DNA were generated from 44 specimens of *H. corynosoma* and 76 of *S. hispida* distributed sympatrically in 6 biogeographic provinces of Mexico with the objective of characterizing and comparing the population genetic structure of 2 acanthocephalan species with opposing life strategies. The phylogeographic studies indicated that the populations of both species lacked a phylogeographic structure and exhibited high haplotype diversity, low nucleotide diversity and low *F*_st_ values among the biogeographic provinces; in combination with negative values on the neutrality test, this suggests that the populations of acanthocephalans are expanding. Paratenic hosts are key for the transmission from intermediate to definitive hosts in the generalist species. However, the inclusion of paratenic hosts does not play a principal role in the population genetic structure of *S. hispida* within its distribution along the coasts of Mexico.

## Graphical abstract

**Figure:**
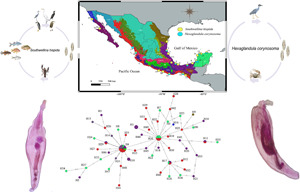

## Introduction

Parasitism is a highly successful lifestyle and has evolved independently at least 60 times in different groups of metazoans worldwide (Price, 1980; Poulin and Morand, 2004). Parasites have been traditionally divided into 2 major groups depending on their life cycle: generalists and specialists (Thompson, 1994, 2005). The generalist parasites use a wide range of definitive hosts, whereas the specialist parasites use a single definitive host to complete their life cycle. Under these 2 opposing strategies, generalist parasites infect a broad spectrum of hosts resulting in an optimal or suboptimal level of fitness, whereas specialist parasites prioritize a single optimal host in which fitness is maximized (Rigaud *et al*., 2010; Lievens *et al*., 2018). Some studies have suggested that parasite life cycle complexity (generalists *vs* specialists) could influence population genetic structure (Nadler, 1995; Criscione and Blouin, 2004; Barrett *et al*., 2008; Archie and Ezenwa, 2011). According to Li *et al*. (2014), a specialist parasite shows significantly less genetic flow; therefore, populations are less connected and are subdivided into smaller populations, leading to strong genetic differentiation. In some cases, the populations might experience bottlenecks, decreasing the effective population size. Moreover, specialist species are more sensitive to stochastic fluctuations that can cause local extinction. In contrast, a generalist parasite shows a high effective population size, high genetic flow and a population that is structured or panmictic. Moreover, a generalist parasite may show greater persistence of populations over the long term because the generalist may be less sensitive to stochastic fluctuations in any given resource as it is able to replace a scarce resource with another (see Sehgal *et al*., 2001; Brant and Ortí, 2003; Archie and Ezenwa, 2011; Li *et al*., 2014).

The recent application of molecular markers has helped establishing a more robust classification scheme in acanthocephalans (Near *et al*., 1998; García-Varela *et al*., 2000). In particular, cytochrome c oxidase subunit 1 (*cox1*) from mitochondrial DNA is among the most useful molecular markers for defining, recognizing and delineating species and better understanding the population genetic structure in acanthocephalans (Steinauer *et al*., 2007; Rosas-Valdez *et al*., 2012, 2020; Alcántar-Escalera *et al*., 2013; Goulding and Cohen, 2014; Perrot-Minnot *et al*., 2018; Pinacho-Pinacho *et al*., 2018; García-Varela *et al*., 2021; Sereno-Uribe *et al*., 2022). Polymorphidae (Meyer, 1931) is an emblematic group of obligate endoparasites with complex life cycles that use vertebrates (marine mammals, fish-eating birds and waterfowl) as definitive hosts and invertebrates (amphipods, decapods and euphausiids) as intermediate hosts to complete their life cycle (Schmidt, 1985; Hoberg, 1986; Pichelin *et al*., 1998; Nickol *et al*., 1999, 2002; Kennedy, 2006). Currently, the family is classified into 14 genera with approximately 129 species (Schmidt, 1973; Dimitrova and Georgiev, 1994; Nickol *et al*., 1999, 2002; Aznar *et al*., 2006; Amin, 2013; García-Varela *et al*., 2013*b*; Presswell *et al*., 2020). Phylogenetic analyses based on multiple molecular markers have suggested that the family is monophyletic as are the genera *Hexaglandula* (Petrochenko, 1950) and *Southwellina* (Witenberg, 1932) (see García-Varela *et al*., 2011, 2013*b*).

Members of the Polymorphidae form a monophyletic group that contains generalist and specialist species. Therefore, this family represents an interesting system to explore and compare the population genetic structure of species with 2 opposing life history strategies. *Hexaglandula corynosoma* (Travassos, 1915) is a specialist species that has been recorded as adult only in the intestine of the yellow-crowned night-heron (*Nyctanassa violacea*) (Linnaeus, 1758), and cystacanths have been recorded in 2 decapod species [the fiddler crabs *Leptuca spinicarpa* (Rathbun) and *Minuca rapax* (Smith)], which serve as intermediate hosts in the Americas (Nickol *et al*., 2002; Guillén-Hernández *et al*., 2008; García-Prieto *et al*., 2010). In contrast, the acanthocephalan *Southwellina hispida* (Van Cleave, 1925) Witenberg, 1932 is a generalist species and is considered one of the most abundant species of polymorphids associated with piscivorous birds throughout the world (see Amin *et al*., 2022). In Mexico, *S. hispida* has been documented in 13 piscivorous bird species (García-Prieto *et al*., 2010; García-Varela *et al*., 2012), and the cystacanth has been recorded in the red swamp crayfish *Procambarus clarkii* (Girard) in the USA (Font, 2007), which serves as an intermediate host that is ingested by several vertebrates that act as paratenic hosts such as frogs and freshwater and brackish fishes (Schmidt, 1985). The paratenic hosts must be ingested by piscivorous birds to complete their life cycle (Schmidt, 1985).

In the current study, we examined the sequences of *cox1* from mitochondrial DNA of 2 closely related polymorphid species, a specialist species (*H. corynosoma*) and a generalist species (*S. hispida*), distributed sympatrically in 6 biogeographic provinces (California, Baja California, Pacific Lowlands, Tamaulipas, Veracruzan and the Yucatán Peninsula) of Mexico with the objective of characterizing and comparing the population genetic structure of 2 acanthocephalan species with opposing life history strategies.

## Materials and methods

### Specimen collection

**Fig. 1.**
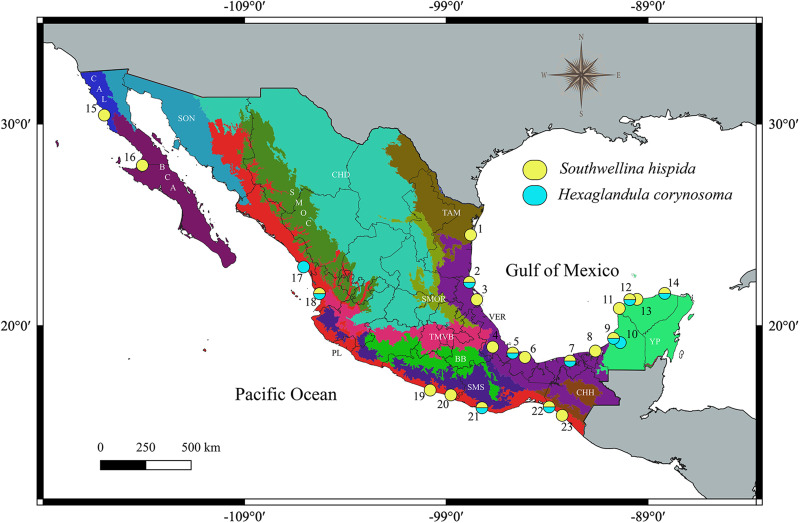
Map of Mexico showing the sampled sites for the birds. Localities with a circle of yellow and turquoise colour were positive for the infection with *Southwellina hispida* and *Hexaglandula corynosoma*, respectively; localities correspond to those in Table 1.

A total of 75 birds, and 36 fishes, were collected between October 2006 and December 2021 in 23 localities across 6 biogeographic provinces (California, Baja California, Pacific Lowlands, Tamaulipas, Veracruzan and the Yucatán Peninsula) of Mexico (Table 1; Fig. 1). Birds and fishes were dissected within the following 4 h, and their viscera were placed in separate Petri dishes containing a 0.75% saline solution and examined under a dissecting microscope. The acanthocephalans recovered were washed in 0.75% saline solution and placed overnight in distilled water at 4°C and subsequently preserved in 100% ethanol. Birds and fishes were identified using the field guide of Howell and Webb (1995) and Miller *et al*. (2005), respectively.

**Table 1.** Specimens’ information, collection sites (CS), host name; number of host examined/infected (prevalence of infection); number of specimens analysed (*n*); locality, geographical coordinates, GenBank accession number for specimens studied in this study

						Coordinates		GenBank
CS	State	Species	Host	*n*	Locality	North	West	*Cox1*
	Tamaulipas							
1		*Southwellina hispida*	*Pelecanus occidentalis* (5/1)	1	Laguna Madre, Tamaulipas	24°29′00″	97°45′00″	FJ824179
	Veracruz							
2		*Hexaglandula corynosoma*	*Nyctanassa violacea* (2/2)	5	Rivera, Veracruz	22°06′54.4″	97°46′37.6″	**OQ152151–155**
		*S. hispida*	*Ardea alba* (1/1)	1	Rivera, Veracruz			**OQ152190**
3		*S. hispida*	*Thalasseus maximus* (5/3)	5	Tamiahua, Veracruz	21°15′49″	97°27′41″	**OQ152203-207**
4		*S. hispida*	*Nycticorax nycticorax* (4/3)	1	Los Chivos, Veracruz	18°56′13″	95°58′08″	FJ824173
5		*H. corynosoma*	*N. violacea* (8/6)	6	Tlacotalpan, Veracruz	18°36′00″	95°39′00″	EF467869 **OQ152124-128**
		*S. hispida*	*Tigrisoma mexicanum* (1/1)	1	Tlacotalpan, Veracruz			EF467867 **OQ152164-166**
		*S. hispida*	*A. alba* (1/1)	1	Tlacotalpan, Veracruz			HM636468
		*S. hispida*	*Nannopterum brasilianus* (7/5)	4	Tlacotalpan, Veracruz			HM636469 **OQ152167-169**
		*S. hispida*	*Rupornis magnirostris* (2/2)	2	Tlacotalpan, Veracruz			**OQ152218-219**
		*S. hispida*	*Cichlasoma urophthalmums* (10/5)	1	Tlacotalpan, Veracruz			FJ824172
6		*H. corynosoma*	*N. violacea* (1/1)	5	Catemaco, Veracruz	18°24′21.6″	95°6′7.2″	**OQ152139-143**
		*S. hispida*	*N. nycticorax* (4/3)	1				FJ824173
	Tabasco							
7		*H. corynosoma*	*N. violacea* (1/1)	1	Espino, Tabasco	18°14′47″	92°49′57″	EU189486
		*S. hispida*	*Egretta garzetta* (1/1)	1	Espino, Tabasco			EF467868
	Campeche							
8		*S. hispida*	*Egretta tricolor* (1/1)	1	Laguna de Términos, Campeche	18°44′01″	91°34′50″	FJ824174
9		*H. corynosoma*	*N. violacea* (1/1)	4	Champoton, Campeche	19°16′50″	90°36′51″	**OQ152156-159**
		*S. hispida*	*Pandion haliaetus* (1/1)	1	Champoton, Campeche			**OQ152208**
10		*H. corynosoma*	*N. violacea* (1/1)	3	Ulumal, Campeche	19°17′50″	90°37′15″	**OQ152144-146**
	Yucatán							
11		*S. hispida*	*A. alba* (1/1)	5	Celestún, Yucatán	20° 50′ 53.5″	90° 24′ 22″	FJ824175 **OQ152170-173**
		*S. hispida*	*C. urophthalmum* (6/4)	1	Celestún, Yucatán			FJ824176
12		*H. corynosoma*	*Leptuca spinicarpa*	1	Chuburna, Yucatán	21°13′18″	89°49′44″	EU189485
		*S. hispida*	*Egretta caerulea* (1/1)	6	Chuburna, Yucatán			**OQ152191-196**
13		*S. hispida*	*C. urophthalmum* (10/5)	5	Progreso, Yucatán	21°16′24.2″	89°39′34.9″	FJ824178 **OQ152186-189**
14		*H. corynosoma*	*N. violacea* (1/1)	6	Ría Lagartos, Yucatán	21°35′32″	88°09′48″	EU189487 **OQ152129-133**
		*S. hispida*	*C. urophthalmum* (5/4)	1	Ría Lagartos, Yucatán			FJ824177
	Baja California Norte							
15		*S. hispida*	*Botaurus lentiginosus* (1/1)	1	Bahía de San Quintin, Baja California Norte	30°24′57″	115°54′72″	FJ824189
	Baja California Sur							
16		*S. hispida*	*Nannopterum auritus* (3/1)	3	Guerrero, Negro, Baja California Sur	27°57′32″	114°03′22″	**OQ152180-181** FJ824187
		*S. hispida*	*N. nycticorax* (1/1)	3	Guerrero, Negro, Baja California Sur			FJ824188 **OQ152182-183**
	Sinaloa							
17		*H. corynosoma*	*N. violacea* (1/1)	4	Caimanero, Sinaloa	22°53′4.65″	106°03′39.15″	**OQ152147-150**
	Nayarit							
18		*H. corynosoma*	*N. violacea* (1/1)	6	La Tovara, Nayarit	21°32′43.66″	105°16′24.12″	EU189488 **OQ152134-138**
		*S. hispida*	*N. brasilianus* (2/1)	1	La Tovara, Nayarit			FJ824185
		*S. hispida*	*Anhinga anhinga* (3/1)	2	La Tovara, Nayarit			**OQ152174** FJ824186
		*S. hispida*	*Butorides virescens* (2/1)	3	La Tovara, Nayarit			**OQ152175-176** FJ824184
		*S. hispida*	*Ardea herodias* (2/1)	3	La Tovara, Nayarit			**OQ152177-178** FJ824183
		*S. hispida*	*T. mexicanum* (1/1)	2	La Tovara, Nayarit			**OQ152179** FJ824182
	Guerrero							
19		*S. hispida*	*Cichlasoma trimaculatum* (5/3)	3	Tres Palos, Guerrero	16°48′00″	99°47′00″	FJ824180 **OQ152184-185**
		*S. hispida*	*N. brasilianus* (1/1)	1	Tres Palos, Guerrero			FJ824181
20		*S. hispida*	*N. nycticorax* (1/1)	1	Marquelia, Guerrero	16°33′19.75″	98°48′38.89″	**OQ152217**
	Oaxaca							
21		*H. corynosoma*	*N. violacea* (1/1)	1	Laguna Manialtepec, Oaxaca	15°56′25″	97°11′40″	**OQ152123**
		*S. hispida*	*A. alba* (1/1)	4	Laguna Manialtepec, Oaxaca			**OQ152197-200**
		*S. hispida*	*N. brasilianus* (2/1)	2	Laguna Manialtepec, Oaxaca			**OQ152201-202**
	Chiapas							
22		*H. corynosoma*	*N. violacea* (1/1)	4	Tonala, Chiapas	15°58′20″	93°51′23″	**OQ152160-163**
		*S. hispida*	*A. alba* (2/2)	5	Tonala, Chiapas			**OQ152212-216**
23		*S. hispida*	*N. nycticorax* (1/1)	3	Pijijiapan, Chiapas	15°33′02″	93°15′53″	**OQ152209-211**

The sample number for each locality corresponds with the same number in Fig. 1. Sequences in bold were generated in the current study.

### Morphological analyses

Selected adult acanthocephalans were gently punctured with a fine needle in the trunk, stained with Mayer's paracarmine, destained in 70% acid ethanol, dehydrated in a graded ethanol series, cleared in methyl salicylate and mounted in Canada balsam. Specimens were examined using a compound microscope Leica DM 1000 LED equipped with bright field (Leica, Wetzlar, Germany). The acanthocephalans were identified by conventional morphological criteria following Petrochenko (1958). In addition, descriptions of *H. corynosoma* and *S. hispida* were consulted as needed (Schmidt, 1973; Nickol *et al*., 2002; Amin *et al*., 2022). For scanning electron microscopy, 2 adult specimens of each species were dehydrated with an ethanol series, critical point dried, sputter coated with gold and examined with a Hitachi Stereoscan Model S-2469N scanning electron microscope operating at 15 kV from the Instituto de Biología, Universidad Nacional Autónoma de México (UNAM). Adult specimens were deposited in the Colección Nacional de Helmintos, under numbers CNHE: 11823 and 11824, Instituto de Biología, Universidad Nacional Autónoma de México, Mexico City

### DNA isolation, amplification and sequencing

A total of 97 specimens, 41 identified as *H. corynosoma* and 56 as *S. hispida* were placed individually in tubes and digested overnight at 56°C in a solution containing 10 mm Tris–HCl (pH 7.6), 20 mm NaCl, 100 mm Na_2_-EDTA (pH 8.0), 1% Sarkosyl and 0.1 mg mL^−1^ proteinase K. Following digestion, DNA was extracted from the supernatant using the DNAzol reagent (Molecular Research Center, Cincinnati, Ohio, USA) according to the manufacturer's instructions. The *cox1* of the mitochondrial DNA was amplified using the forward primer 5′-AGTTCTAATCATAA(R)GATAT(Y)GG-3′ and reverse primer 5′-TAAACTTCAGGGTGACCAAAAAATCA-3′ (Folmer *et al*., 1994). Polymerase chain reactions (PCRs) (25 *μ*L) consisted of 10 *μ*L of each primer, 2.5 *μ*L of 10× buffer, 2 mm MgCl_2_ and 1 U of Taq DNA polymerase (Platinum Taq, Invitrogen Corporation, São Paulo, Brazil). PCR cycling parameters for the molecular marker consisted of denaturation at 94°C for 1 min, 35 cycles of 94°C for 1 min, 40°C for 1 min and 72°C for 1 min, followed by a post-amplification incubation at 72°C for 10 min. Sequencing reactions were performed using ABI Big Dye (Applied Biosystems, Boston, Massachusetts, USA) terminator sequencing chemistry, and reaction products were separated and detected using an ABI 3730 capillary DNA sequencer. Contigs were assembled and base-calling differences resolved using Codoncode Aligner version 9.0.1 (Codoncode Corporation, Dedham, Massachusetts, USA) and submitted to the GenBank dataset (Table 1).

### Alignments, population genetic structure and historical demographic

Newly obtained sequences in the current research of *S. hispida* were aligned with 22 other sequences of *S. hispida* (EF467867–868, HM636468–469, FJ824172–189) downloaded from GenBank (Table 1), forming a dataset of 78 sequences with 646 characters, and the new sequences of *H. corynosoma* were aligned with other sequences of *H. corynosoma* (EU189485–486, EU189488 and EF467869), downloaded from GenBank (Table 1), forming a dataset of 46 sequences with 644 characters. Sequences of each dataset were aligned separately using software ClustalW with default parameters implemented in MEGA version 7.0 (Kumar *et al*., 2016).

To analyse the molecular information in the framework of population genetics, we grouped individuals of *H. corynosoma* and *S. hispida* into populations considering the biogeographic provinces (California, Baja California, Pacific Lowlands, Tamaulipas, Veracruzan and the Yucatán Peninsula). Intrapopulation variation was summarized using standard statistics: number of haplotypes (*H*), number of segregating sites (*S*), haplotype diversity (*H*_d_), nucleotide diversity (*P*_i_) and average number of nucleotide differences (*K*), were all calculated using the program DnaSP v.5.10 (Rozas *et al*., 2003). To examine haplotype frequency among the populations of *H. corynosoma* and *S. hispida* a statistical network was constructed independently, using the program PopART with the median joining algorithm (Bandelt *et al*., 1999). The degree of genetic differentiation among the populations was estimated using the fixation indices *F*_st_ (Hudson *et al*., 1992), with the program Arlequin v.3.5 (Excoffier and Lischer, 2010). To investigate the genetic variation among populations or within populations, the analysis of molecular variance was performed, considering genetic distance among the haplotypes using Arlequin v.3.5. To investigate the population history and demography, Tajima's *D* (Tajima, 1989) and Fu's *F*_s_ (Fu, 1997) were calculated using DnaSP v.5.10 (Rozas *et al*., 2003). The values were considered significant when the *P* values were less than 0.05.

## Results

### Morphological identification

**Fig. 2.**
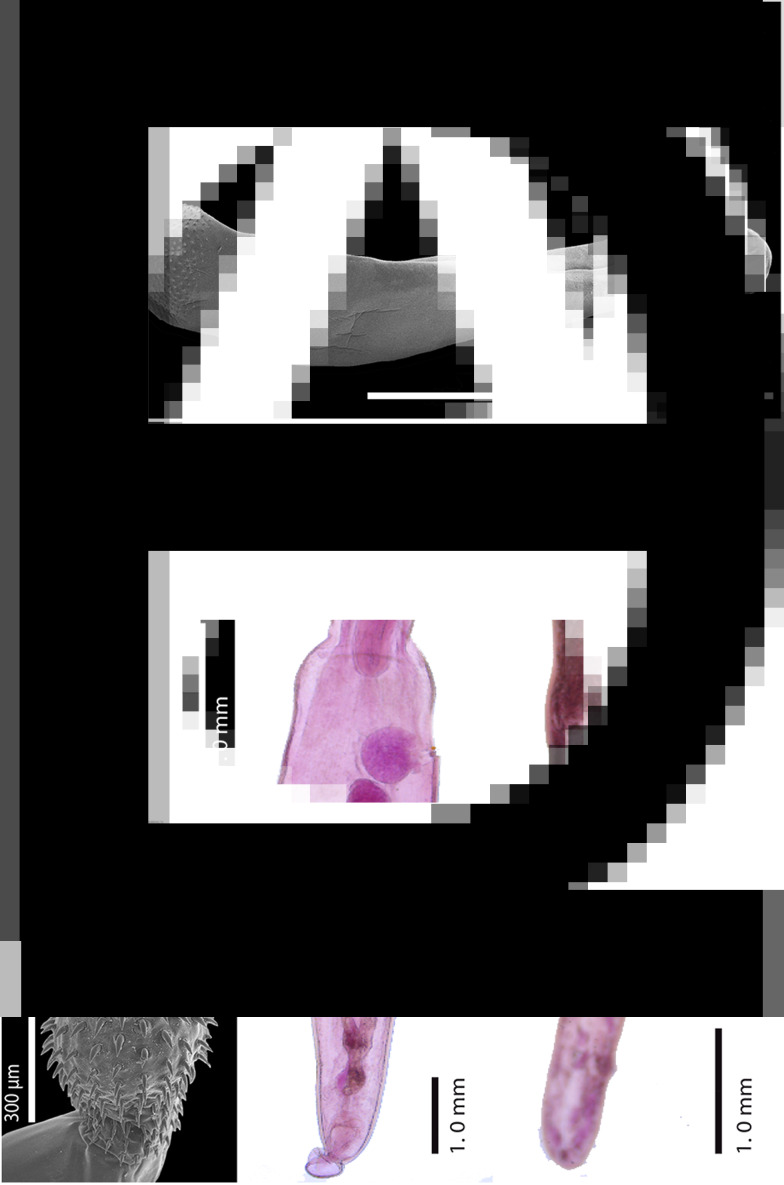
Scanning electron micrographs and photomicrographs of *S. hispida* from *Ardea alba* from Tonala, Chiapas, Mexico (locality 22 in Fig. 1 and Table 1): adult male, whole worm (A); male anterior region (B); proboscis (C); adult male, whole worm (D) and adult female, whole worm (E).

**Fig. 3.**
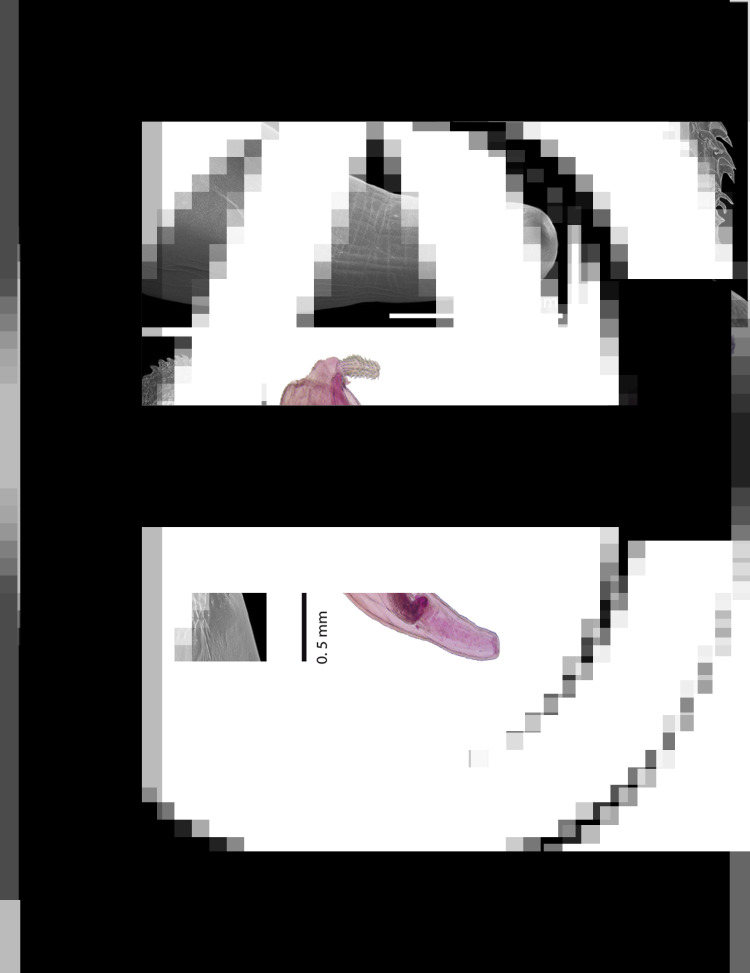
Scanning electron micrographs and photomicrographs of *H. corynosoma* from *Nyctanassa violacea* from La Tovara Nayarit, Mexico (locality 18 in Fig. 1 and Table 1): adult male, whole worm (A); proboscis (B); male anterior region (C); adult male, whole worm (D) and adult female, whole worm (E).

The acanthocephalans recovered from diverse definitive hosts such as herons, gulls, cormorants, pelicans and hawks on both coasts of Mexico show similar morphological characteristics compared with those assigned to *S. hispida* by García-Varela *et al*. (2012) and Amin *et al*. (2022), including (i) an elongated cylindrical trunk with 2 fields of somatic spines on the anterior region of the trunk, (ii) a cylindrical proboscis with a swollen region, (iii) proboscis hooks arranged in 16–17 longitudinal rows of 12–15 hooks per row, (iv) a double-walled proboscis receptacle and (v) 4 tubular cement glands in males (Fig. 2A–E). The acanthocephalans recovered from the intestine of the yellow-crowned night-heron (*N. violacea*) in coastal Mexico show morphological characteristics that match those assigned to *H. corynosoma* by Nickol *et al*. (2002), Guillén-Hernández *et al*. (2008) and Amin *et al*. (2022), including (i) an elongate cylindrical trunk, swollen anteriorly with a single field of somatic spines in the anterior region of the trunk; (ii) a cylindrical proboscis; (iii) proboscis hooks arranged in 16 longitudinal rows of 11–12 hooks per row; (iv) a double-walled proboscis receptacle and (v) 6 tubular cement glands in males (Fig. 3A–E).

### Population genetic structure and demographic analysis

The mitochondrial marker was successfully amplified for 56 *S. hispida* individuals and 41 *H. corynosoma* individuals. The complete alignment of the *cox1* dataset contained 78 *S. hispida* individuals with a total length of 646 bp, whereas the *cox1* dataset of *H. corynosoma* contained 46 individuals with a total length of 644 bp. No insertions or deletions were detected in any of the sequences, and when the sequences were translated into proteins, no stop codons were found.

**Fig. 4.**
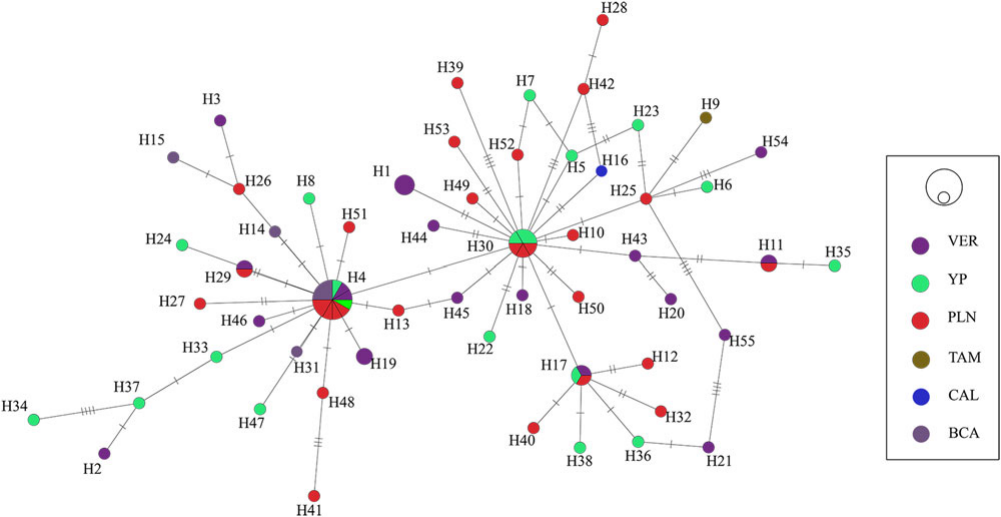
Haplotype network of samples of *S. hispida*, built with the gene *cox1* from mitochondrial DNA. Each circle represents a haplotype, with size proportional to the haplotype's frequency in the populations. Mutational steps are symbolized by dashes. Biogeographic provinces: Veracruzan (VER); Yucatán Peninsula (YUC); Pacific Lowlands (PLN); Tamaulipas (TAM); California (CAL) and Baja California (BCA).

**Fig. 5.**
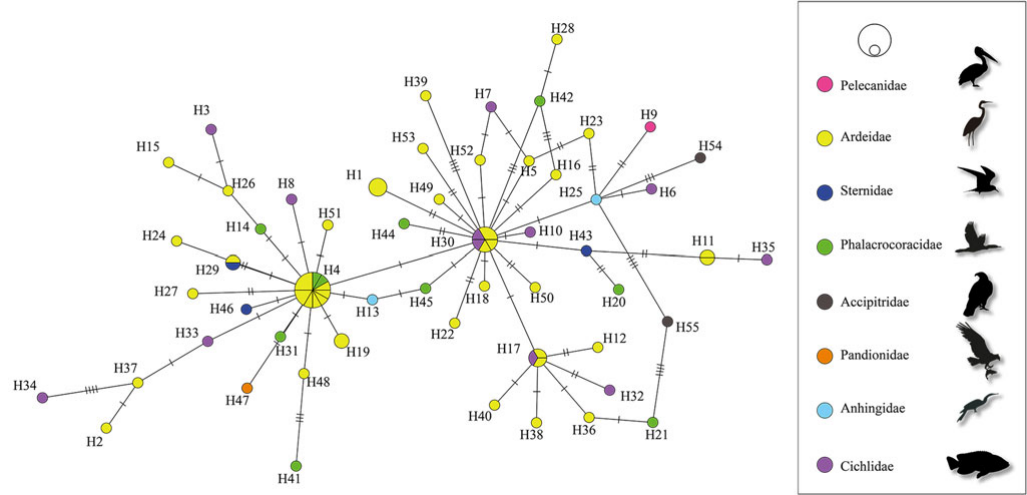
Host haplotype network of samples of *S. hispida*, built with the gene *cox1* from mitochondrial DNA. The paratenic and definitive hosts were grouped by families. Each circle represents a haplotype, with size proportional to the haplotype's frequency in the populations. Mutational steps are symbolized by dashes.

The haplotype network built for the generalist species *S. hispida* did not show a phylogeographic structure; in fact, of the 55 mtDNA haplotypes detected, 50 were unique (singlets), and 5 others were shared haplotypes (H4, H11, H17, H29 and H30). The most frequent haplotype (H4, *n* = 12) corresponded to specimens from 4 biogeographic provinces (Pacific Lowlands, Baja California, Veracruzan and the Yucatán Peninsula). Most of the identified haplotypes were separated from one another by 1, 2, 3 or up to 5 substitutions (see Fig. 4). The haplotype diversity was very high (*H*_d_ = 0.970) and nucleotide diversity was low (*p*_i_ = 0.00555) among the populations from the 4 biogeographic provinces sampled (Pacific Lowlands, Baja California, Veracruzan and the Yucatán Peninsula) of Mexico. Neutrality tests (Tajima's *D*, −2.473 and Fu's *F*_s_, −34.252) were negative for all regions (see Table 2), indicating an excess of rare alleles greater than what would be expected under neutrality and suggesting a recent population expansion of *S. hispida*. The *F*_st_ values were estimated to assess genetic differentiation among populations from the 4 biogeographic provinces analysed (Pacific Lowlands, Baja California, Veracruzan and the Yucatán Peninsula). Despite the large geographic distances, the *F*_st_ values were low, ranging from −0.007 to 0.058 (Table 3), which indicates that the populations were poorly genetically differentiated from one another. The sampled definitive and paratenic host species of *S. hispida* were grouped into 8 families (Fig. 5). The host haplotype network did not show a pattern; 30 haplotypes were found in herons scattered throughout the network. In the paratenic hosts sampled (cichlid fishes), 11 haplotypes were detected (H3, H6, H7, H8, H10, H17, H30, H32, H33, H34 and H35), suggesting that the paratenic hosts are able to harbour and transmit diverse haplotypes to definitive hosts (Fig. 5).

**Table 2.** Molecular diversity indices and neutrality tests calculated for *cox1* datasets among the populations of *S. hispida* used in this study

Biogeographic provinces	*n*	*S*	*H*	*H*_d_ ± s.d.	*P*_i_ ± s.d.	*K*	Tajima's *D*	Fu's *F*_s_
Veracruzan	21	29	18	0.981 ± 0.023	0.00663 ± 0.00064	4.285	−1.811	−13.228
Yucatán Peninsula	19	26	17	0.988 ± 0.021	0.00607 ± 0.00083	3.918	−1.859	−13.746
Baja California	7	7	5	0.857 ± 0.137	0.00310 ± 0.00095	2.000	−1.553	−1.548
Pacific Lowlands	31	33	23	0.955 ± 0.026	0.00488 ± 0.00064	3.152	−2.282	−21.079
All	78	67	55	0.970 ± 0.012	0.00555 ± 0.00041	3.587	−2.473	−34.252

*n*, number of sequences; *H*, number of haplotypes; *S*, number of segregating sites; *H*_d_, haplotype diversity; *P*_i_, nucleotide diversity; *K*, average number of nucleotide differences.

**Table 3.** Pairwise *F*_st_ values estimated for *cox1*

	VER	YP	BCAL	PL
VER	–			
YP	−0.007	–		
BCAL	0.088	0.082	–	
PL	0.010	0.005	0.058	–

VER, Veracruzan; YP, Yucatan Peninsula; BCAL, Baja California; PL, Pacific Lowlands.

*F*_st_ average among 3 biogeographic provinces = 0.03672.

Significance level = 0.05.

**Fig. 6.**
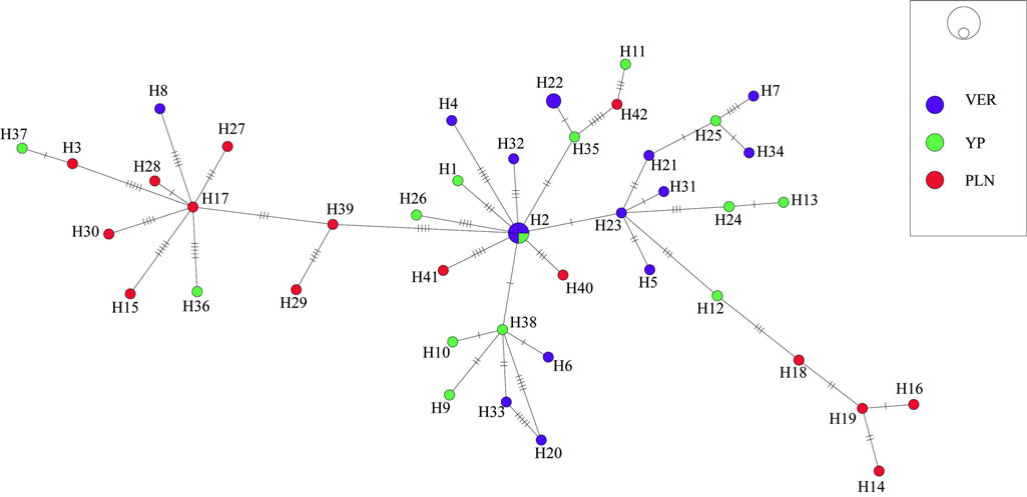
Haplotype network of samples of *H. corynosoma*, built with the gene *cox1* from mitochondrial DNA. Each circle represents a haplotype, with size proportional to the haplotype's frequency in the populations. Mutational steps are symbolized by dashes. Biogeographic provinces: Veracruzan (VER); Yucatán Peninsula (YUC) and Pacific Lowlands (PLN).

The haplotype network of the specialist species *H. corynosoma* did not show a phylogeographic structure for the 42 mtDNA haplotypes detected; 41 were unique (singlets), and only 1 was shared (H2, *n* = 4) between 2 biogeographic provinces (Veracruzan and the Yucatán Peninsula). Most of the identified haplotypes were separated by several substitutions (see Fig. 6). The haplotype diversity (*H*_d_ = 0.993) was very high and nucleotide diversity was low (*p*_i_ = 0.01166) among the populations from the 3 biogeographic provinces sampled (Pacific Lowlands, Veracruzan and the Yucatán Peninsula). Neutrality tests (Tajima's *D*, −1.875 and Fu's *F*_s_, −33.964) were negative for all regions (see Table 4), which indicates an excess of rare alleles greater than what would be expected under neutrality, suggesting a recent population expansion of *H. corynosoma*. The *F*_st_ values were estimated to assess genetic differentiation among the populations from the 3 biogeographic provinces analysed (Pacific Lowlands, Veracruzan and the Yucatán Peninsula). Despite the large geographic distances, the *F*_st_ values were low, ranging from −0.019 to 0.059 (Table 5), which indicates that the populations were poorly genetically differentiated from one another.

**Table 4.** Molecular diversity indices and neutrality tests calculated for *cox1* datasets among the populations of *H. corynosoma* used in this study

Biogeographic provinces	*n*	*S*	*H*	*H*_d_ ± s.d.	*P*_i_ ± s.d.	*K*	Tajima's *D*	Fu's *F*_s_
Veracruzan	17	32	14	0.971 ± 0.032	0.00884 ± 0.00132	5.691	−1.707	−6.056
Yucatán Peninsula	14	38	14	1.0 ± 0.027	0.01165 ± 0.00177	7.505	−1.613	−8.104
Pacific Lowlands	15	35	15	1.0 ± 0.024	0.01344 ± 0.00114	8.657	−0.926	−8.273
All	46	66	42	0.993 ± 0.007	0.01166 ± 0.00083	7.510	−1.875	−33.964

*n*, number of sequences; *H*, number of haplotypes; *S*, number of segregating sites; *H*_d_, haplotype diversity; *P*_i_, nucleotide diversity; *K*, average number of nucleotide differences.

**Table 5.** Pairwise *F*_st_ values estimated for *cox*

	VER	YP	PL
VER	–		
YP	−0.019	–	
PL	0.112	0.059	–

VER, Veracruzan; YP, Yucatan Peninsula; PL, Pacific Lowlands

*F*_st_ average among 3 biogeographic provinces = 0.05609.

Significance level = 0.05.

## Discussion

To the best of our knowledge, *S. hispida* and *H. corynosoma* are 2 species that use piscivorous birds as definitive hosts and decapods as intermediate hosts, share a common ancestor within Polymorphidae and are distributed sympatrically along the coastline of Mexico (Guillén-Hernández *et al*., 2008; García-Prieto *et al*., 2010; García-Varela *et al*., 2013*b*). The generalist species *S. hispida* was described in Japan by Van Cleave in 1925, and since then it has been recorded in the body cavities of fishes and reptiles as a cystacanth (larval stage) and as an adult form in the intestines of diverse piscivorous birds in Asia, Europe and the Americas (see Amin *et al*., 2022). In Mexico, adults of *S. hispida* have been recorded in 15 piscivorous bird species (García-Prieto *et al*., 2010; García-Varela *et al*., 2012). It is well known that helminths with a broad spectrum of definitive hosts show phenotypic plasticity in their morphological traits. Schmidt (1973), García-Varela *et al*. (2012) and Amin *et al*. (2022) documented the phenotypic plasticity of *S. hispida* recovered from diverse definitive hosts including in traits such as body size, leminisci, proboscis receptacle, testes, cement glands and the reproductive system in females. However, diagnostic characters such as proboscis shape, number of proboscis hooks and the presence of 2 fields of spines on the anterior trunk region in both sexes did not vary among specimens, including our specimens collected from the coasts of the Gulf of Mexico and the Pacific Ocean slopes (see Fig. 2A–E). In Brazil, the specialist species *H. corynosoma* was described as parasite of the yellow-crowned night heron (*N. violacea*) by Travassos in 1915. Since then, *H. corynosoma* has been recorded in Puerto Rico, the USA and Mexico in the same definitive host species (Cable and Quick, 1954; Nickol *et al*., 2002; Guillén-Hernández *et al*., 2008). Our specimens identified as *H. corynosoma* agree morphologically with those previously described by Nickol *et al*. (2002) and Guillén-Hernández *et al*. (2008). For example, an elongated trunk, swollen anteriorly; covered with spines on the anterior region of the trunk; a cylindrical proboscis with 16 longitudinal rows of 11–12 hooks each; a conical neck; ovoid testes located in the swollen portion of the trunk and long, tubular cement glands (see Fig. 3A–E).

The intraspecific genetic divergence estimated in the current study among the 78 isolates of *S. hispida* and the 46 isolates of *H. corynosoma* ranged from 0.00 to 1.5% and from 0.00 to 2.6%, respectively. These values of intraspecific genetic divergence are similar to those previously reported for isolates of polymorphid species such as *Andracantha sigma* (Presswell *et al*., 2017) recovered from 3 definitive hosts, the Otago shag, *Leucocarbo chalconotus* (Gray), spotted shag *Phalacrocorax punctatus* (Sparrman) and Otago little blue penguin, *Eudyptula novaehollandiae* (Forster) from New Zealand, which ranged from 0.00 to 0.32% (Presswell *et al*., 2017); similar to those among 14 adults, 3 acanthella and 4 cystacanths of *Pseudocorynosoma constrictum* (Van Cleave, 1918) Aznar *et al*. (2006) recovered from 7 wild duck species and the freshwater amphipod *Hyalella azteca* (Saussure) in central Mexico, which ranged from 0.0 to 3.0% (García-Varela *et al*., 2013*a*) and similar to those among 19 adults recovered from 3 fish-eating bird species and 33 cystacanths recovered from 19 freshwater fish species identified as *Polymorphus brevis* (Van Cleave, 1916) Travassos, 1926, which ranged from 0.00 to 1.6% (Alcántar-Escalera *et al*., 2013). Furthermore, the values from this study were also similar to those among adults and cystacanths of *Corynosoma hannae* Zdzitowiecki, 1984 recovered from the New Zealand sea lion (*Phocarctos hookeri* Grey), Stewart Island shag (*L. chalconotus* Gray), spotted shags (*P. punctatus* Sparrman), yellow-eyed penguins (*Megadyptes antipodes* Hombron and Jacquinot), New Zealand brill (*Colistium guntheri* Hutton) and New Zealand sole (*Peltorhamphus novaezeelandiae* Gunther), which ranged from 0.00 to 2.8% (Hernández-Orts *et al*., 2017) and those among *Corynosoma australe* (Johnston, 1937) recovered from the California sea lion (*Zalophus californianus* Lesson), South American sea lions (*Otaria flavescens* Shaw), South American fur seals (*Arctocephalus australis* Zimmermann), Magellanic penguins (*Spheniscus magellanicus* Forster) and cystacanths recovered from marine fishes in Argentina, which ranged from 1.0 to 1.7% (García-Varela *et al*., 2021).

The haplotype network genealogy generated in this study based on *cox1* sequences from *S. hispida* (generalist) and *H. corynosoma* (specialist) did not show a phylogeographic structure; therefore, the haplotypes could not be grouped into their own geographic clusters. In fact, the specimens of *S. hispida* were not correlated with their definitive host family (see Figs 4 and 5). The populations analysed for both species of acanthocephalans were classified into biogeographical provinces separated by geographical barriers as follows: mountains, the dry lowlands of the Isthmus of Tehuantepec, the Balsas Depression and the central Trans-Mexicana Volcanic Belt (Barrier *et al*., 1998; Ferrari *et al*., 2012; Morrone *et al*., 2017). Despite the large geographic distances, the *F*_st_ values estimated among the populations of both species were very low (Tables 3 and 5), indicating that the populations were poorly genetically differentiated from each other; this can be explained by the migration patterns of birds along the coasts of Mexico. Historical events leave signatures in the DNA, and neutrality tests can infer the demographic history of populations. In both species analysed (generalist and specialist), the estimated values of Fu's *F*_s_ and Tajima's *D* among the populations were negative (see Tables 2 and 4). In addition, high haplotype diversity and an excess of low-frequency haplotypes were detected in both networks (Figs 4 and 6), although the haplotypes differed from one another by fewer than 5 nucleotide substitutions. These findings confirmed that both populations of acanthocephalans had experienced rapid population growth in the past.

In this study, we found that 2 species of acanthocephalans with opposing life history strategies (generalist and specialist) showed similar population genetics patterns. This pattern was not consistent with the specialist–generalist variation hypothesis (SGVH), which predicts that: (i) populations of specialists may be less connected and more subdivided into smaller populations than generalists; (ii) specialists are expected to have lower effective population sizes than generalists and to be composed of populations with less gene flow and (iii) generalists will show high effective population sizes, high genetic flow and highly structured populations (see Dennis *et al*., 2011; Li *et al*., 2014). According to the SGVH, the populations of *S. hispida* (generalist species) resemble the specialist model. To date, only a few phylogeographic studies of generalist acanthocephalans have been conducted using *cox1* as a molecular marker. For example, *Profilicollis altmani* (Perry, 1942), which has a broad distribution across North and South America and parasitizes multiple species of intermediate hosts and diverse species of marine birds that act as definitive hosts (gulls, ducks, sanderling and common tern), showed a lack of population genetic structure with high haplotype diversity and low nucleotide diversity, suggesting that *P*. *altmani* have experienced a period of rapid population growth in the past (see Goulding and Cohen, 2014). Similarly, *Profilicollis novaezelandensis* Brockerhoff and Smales (2002) is a parasite that has been recorded as an adult in gulls (*Larus* spp.) and oystercatchers (*Haematopus* spp.) and as cystacanths in the shore crab *Hemigrapsus crenulatus* (Milne-Edwards) distributed along the east coast of New Zealand's South Island (Brockerhoff and Smales, 2002); population genetic analyses from 50 *P. novaezelandensis* individuals from 8 localities showed a lack of population genetic structure with high haplotype diversity and low nucleotide diversity (Hay *et al*., 2018).

The 2 species of acanthocephalans analysed here are considered to be typical components of the helminth fauna of piscivorous birds in the Americas. Of the 19 piscivorous birds recorded as definitive hosts, 11 belong to the family Ardeidae, which represent 58% of the host diversity, suggesting that the ardeids could be the ancestral hosts of *S. hispida* and *H. corynosoma* with secondary and independent colonization events to other piscivorous birds. In particular, *S. hispida* was recorded in diverse piscivorous birds such as pelicans, cormorants, eagles, hawks, anhingas and royal terns with a lower level of infection (prevalence values <20%) than those of ardeid hosts (prevalence values from 80 to 100%), suggesting that these piscivorous birds may act as suboptimal definitive hosts.

The population genetic structures of *S. hispida* and *H. corynosoma* showed similar patterns. However, these species use different strategies to complete their life cycles. The main ecological difference between *S. hispida* and *H. corynosoma* is the inclusion of paratenic hosts in *S. hispida* (fishes from the families Cichlidae, Eleotridae, Characidae, Lutjanidae, Centropomidae, Sparidae, Scianidae and Paralichthyidae) (García-Prieto *et al*., 2010). The paratenic hosts act as a trophic bridge and facilitate the transmission between the intermediate host and definitive host, but apparently do not play a central role in the population genetic structure of *S. hispida* within its distribution along the coasts of Mexico.
